# Epidemiology of distal radius fracture: a regional population-based study in Japan

**DOI:** 10.1186/s12891-023-06608-2

**Published:** 2023-06-13

**Authors:** Jiro Ando, Tsuneari Takahashi, Ryusuke Ae, Takashi Ajiki, Tomohiro Matsumura, Wataru Sasao, Masahiko Abe, Katsushi Takeshita

**Affiliations:** 1 Department of Orthopedics, School of Medicine, Jichi Medical University, Yakushiji 3311-1, Shimotsuke, Tochigi 329-0498 Japan; 2 Department of Orthopedic Surgery, Ishibashi General Hospital, Shimokoyama 1-15-4, Shimotsuke, Tochigi 329-0502 Japan; 3 Division of Public Health, Center for Community Medicine, Jichi Medical University, Yakushiji 3311-1, Shimotsuke, Tochigi 329-0498 Japan; 4 Jichi Medical University Hospital Life Saving Emergency Center, Yakushiji 3311-1, Shimotsuke, Tochigi 329-0498 Japan; 5 Hokkaido Prefecture Haboro Hospital, Hokkaido, Sakaemachi 110, Haborochou, Tomamaegun, Hokkaido 078-4197 Japan

**Keywords:** Distal radius fracture, Epidemiology, Incidence, Japan

## Abstract

**Background:**

Distal radius fracture (DRF) is very common worldwide. In particular, aging countries have numerous patients with DRF, resulting in an urgent need for active preventive measures. As few epidemiological studies have investigated DRF in Japan, we aimed to identify the epidemiological characteristics of patients of all ages with DRF in Japan.

**Methods:**

This descriptive epidemiologic study analyzed data obtained from clinical information of patients diagnosed with DRF from January 1, 2011, to December 31, 2020, at a prefectural hospital in Hokkaido, Japan. We calculated the crude and age-adjusted annual incidences of DRF and described the age-specific incidence, injury characteristics (injury location and cause, seasonal differences, and fracture classification), and 1- and 5-year mortality rates.

**Results:**

A total of 258 patients with DRF were identified, of which 190 (73.6%) were female and the mean age (standard deviation) was 67.0 (21.5) years. The crude annual incidence of DRF ranged from 158.0 to 272.6 per 100,000 population/year, and the age-adjusted incidence among female patients demonstrated a significant decreasing trend during 2011–2020 (Poisson regression analysis; *p* = 0.043). The age-specific incidence differed by sex, with peaks at 10–14 years for males and 75–79 years for females. The most common cause of injury was a simple fall in patients > 15 year of age and sports injuries in patients ≤ 15 years of age. DRFs were most frequently sustained outdoors and were more common in the winter season. In patients > 15 years of age, the proportions of AO/OTA fracture types A, B, and C were 78.7% (184/234), 1.7% (4/234), and 19.6% (46/234), respectively, and 29.1% (68/234) of patients received surgical treatment for DRF. The 1- and 5-year mortality rates were 2.8% and 11.9%, respectively.

**Conclusions:**

Our findings were mostly consistent with previous global studies. Although the crude annual incidence of DRF was relatively high because of recent population aging, the age-adjusted annual incidence among female patients showed a significant decreasing trend during this decade.

## Background

Distal radius fracture (DRF) is one of the most common fractures worldwide. Global studies outside of Japan have reported that the DRF incidence ranges from 100–190 per 100,000 population/year in males and 282–458 per 100,000 population/year in females in the general population [1–11]. The age-specific incidence of DRF has bimodal distribution in both sexes, with the first peak in the teenage years and the second peak after 70 years of age, and the incidence is greater in females than males in the population older than 50 years [1–11]. Worldwide, aging countries have experienced a large number of patients with DRF, resulting in an urgent need for active preventive measures [5–7].

In Japan, DRF has been the focus of epidemiological studies in three regions [12–17]. These epidemiological studies were conducted in Tottori Prefecture during 1986–1988 and 1992–1995 [12], in Sakaiminato City during 2010–2012 [13], and in Sado City during 2004–2006 and 2010–2015 [14–17]. The reported incidence of DRF in Japan ranges from 46–82 per 100,000 population/year in males and 147–432 per 100,000 population/year in females [12, 13]. However, these studies mainly focused on osteoporotic older adults aged 50 years or older [13–16]. In contrast, there is little epidemiological information about DRF in patients of all ages in Japan [17]. Therefore, using regional population-based data, the present study aimed to assess the epidemiological characteristics of patients of all ages with DRF by focusing on the variables of person, time, and place, which are commonly used in descriptive epidemiologic studies.

## Methods

### Design, setting, and participants

This descriptive epidemiologic study analyzed data obtained from clinical information of patients diagnosed with DRF from January 1, 2011, to December 31, 2020, at a prefectural hospital in Hokkaido, Japan. Patients diagnosed with DRF were identified by searching for the International Classification of Diseases, 10th revision (ICD-10) codes S52.5 and S52.6 in the medical records database; each identified patient was carefully reviewed and their data were retrieved from the medical records. We excluded patients with ipsilateral re-fracture. This study was approved by the Jichi Medical University Clinical Research Ethics Committee (approval ID: 21–116). The Ethics Committee waived the need for informed consent because of the retrospective observational nature of the study.

**Fig. 1:**
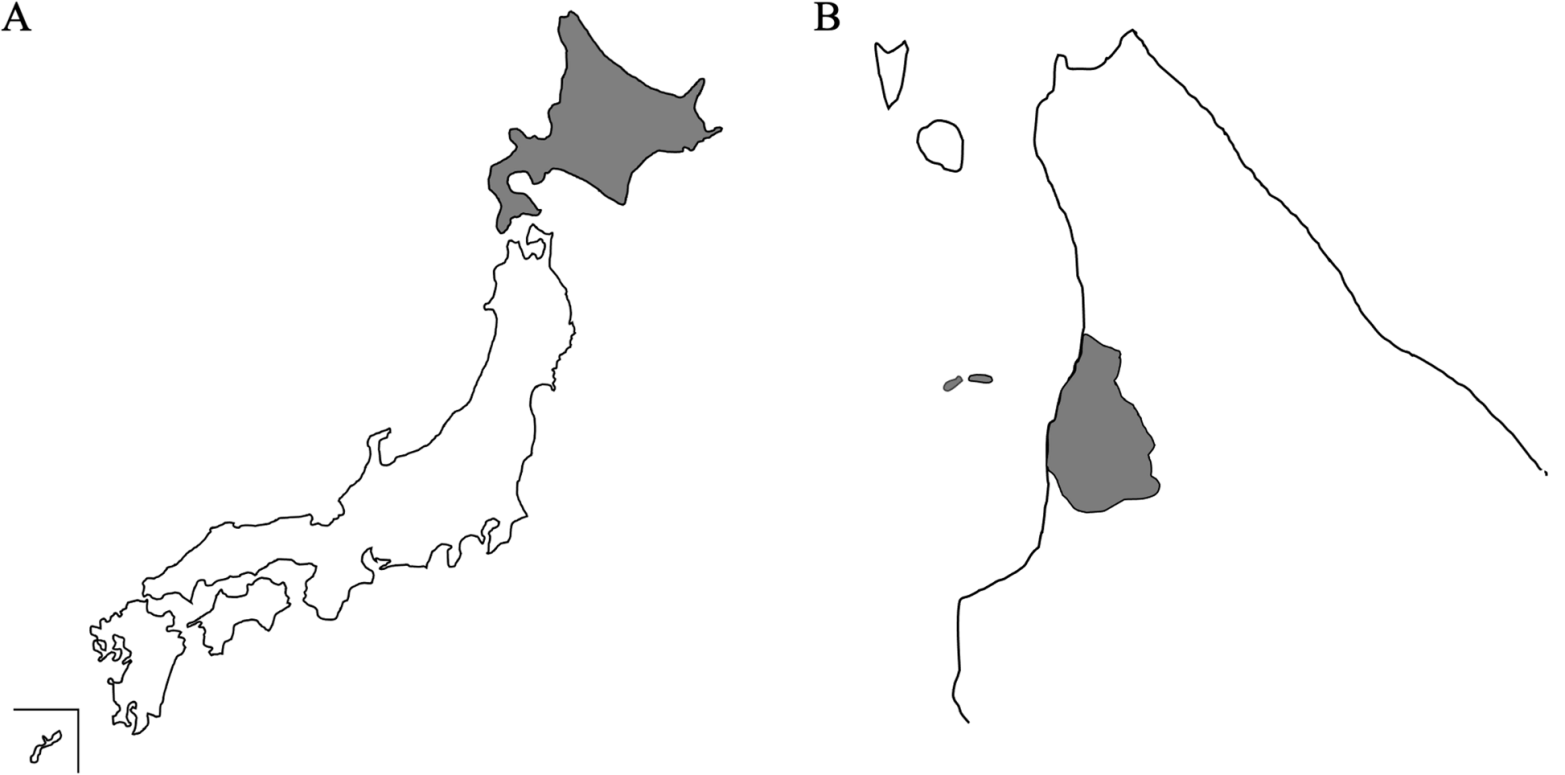
Location of Tomamae district, Hokkaido, Japan. **A** The shaded area shows Hokkaido. **B** Tomamae district is located in northern Hokkaido. Both maps have north at the top

Hokkaido is located in the north of Japan (Fig. 1A), and Tomamae district is a northern region in Hokkaido (44°N) (Fig. 1B) with a cold climate and snowfall from January to March and November to December. According to the government statistics of Japan [18], Tomamae district has an aging and declining population; from 2011 to 2021, the population decreased from 13,155 to 10,772, but the proportion of the population over 65 years old increased from 35.7% to 42.2%. We used registry data obtained from Hokkaido Prefecture Haboro Hospital, which is located in Tomamae district. Hokkaido Prefecture Haboro Hospital is the only hospital in the region that performs orthopedic surgeries. Therefore, almost all patients with fractures within the region are diagnosed and treated by orthopedic surgeons at Hokkaido Prefecture Haboro Hospital, and so the number of fractures diagnosed at this hospital approximates the total number of fractures incurred in the regional population. Patients were eligible for study inclusion if they were diagnosed with DRF at Hokkaido Haboro Prefectural Hospital during 2011–2020.

### Measurements

Demographic information included patient age and sex. Clinical information included injury data (location, cause, and date), fracture data (side, fracture type, use of computed tomography [CT] for diagnosis, and complication of ulnar fracture), treatments, and mortality data. Injury locations were categorized as indoor (e.g., patient's residence, facility) or outdoor (e.g., public space, street). Injury causes were categorized as simple fall, fall from height, traffic accident, crush injury, sports injury, and others. Fracture types were classified according to the Arbeitsgemeinschaft für Osteosynthesefragen Foundation/Orthopaedic Trauma Association (AO/OTA) classification [19], which is the standard classification in orthopedic and injury medicine. Treatment modalities were classified into nonsurgical and surgical treatments. Information on survival and death at 1 year and 5 years after injury was obtained from the medical records.

### Statistical analysis

First, we calculated the annual incidence of DRF from 2011 to 2020, using population data obtained from the government statistics in Japan [18]. The incidence rates were calculated by dividing the annual number of DRF cases by the population in the corresponding year and multiplying by 100,000 (i.e., per 100,000 population/year). Furthermore, to evaluate the incidence of DRF adjusted for the impact of population aging, we calculated age-adjusted annual incidence rates with direct adjustment methods, using the standard population in the year 2000 as the reference. Poisson regression analysis was performed to assess the statistical significance of the annual trends in the age-adjusted incidences of DRF in males and females from 2011 through 2020. Second, the age-specific DRF incidence rates were determined. In this analysis, incidence rates were separately calculated by dividing the 5-year-age-specific number of DRF cases that occurred during 2011–2020 by the corresponding 5-year-age-specific population averaged by summing the populations from 2011 to 2020. Third, we described the distributions of injury location and specific injury causes in accordance with the 5-year age groups. For specific injury causes, we compared the data of patients with DRF aged ≤ 15 years with those aged > 15 years, based on the hypothesis that the injury causes differ between younger and older patients. Fourth, we determined the age and sex distributions by fracture type in patients aged ≤ 15 years compared with patients aged > 15 years. CT use, complications, and treatments were also compared between these groups. Finally, we calculated 1- and 5-year mortality rates using Kaplan–Meier survival curves. Data are presented as the mean and standard deviation (SD) or percentages of each group of patients. The 2.5 and 97.5 percentiles were used to express 95% confidence intervals (CIs). All statistical analyses were performed using EZR software [20].

## Results

A total of 280 patients with DRF were identified. After excluding 20 patients who resided outside Tomamae district and two patients with ipsilateral re-fracture, 258 patients were included in the analysis. There were no patients with simultaneous bilateral fractures and three patients with contralateral DRF injuries at different times in this study period. There were 190 (73.6%) female patients, giving a male-to-female ratio of 1:2.8. The mean (SD) age of the total cohort was 67.0 (21.5) years, ranging from 2 to 99 years; the mean (SD) age was 73.0 (12.9) years for females, and 49.9 (30.4) years for males. Of the 258 patients with DRF, 24 (9.3%) and 175 (67.8%) were ≤ 15 years and ≥ 65 years of age, respectively.

### Incidence of DRF

**Fig. 2:**
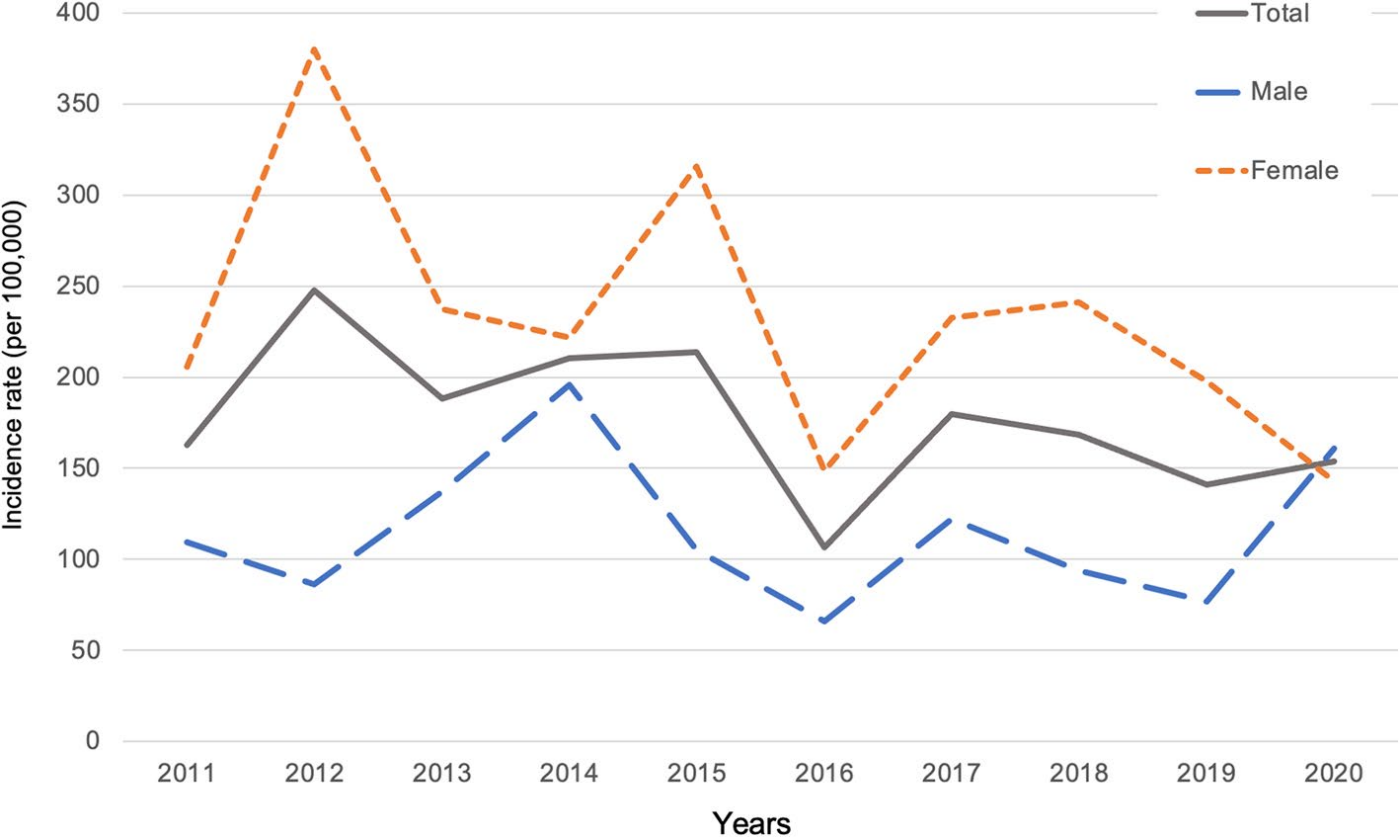
Age-adjusted incidences of distal radial fracture per 100,000 population/year during 2011–2020. Direct adjustment with reference to the standard population in the year 2000

The annual incidence of DRF ranged from 158.0 to 272.6 per 100,000 population/year during 2011–2020, as shown in Table 1. The annual incidence was consistently higher in females than males in all examined years (range, 222.0–429.2 per 100,000 population/year in females; range, 74.0–184.6 per 100,000 population/year in males). The age-adjusted incidence of DRF in females demonstrated a significant decreasing trend from 2011 through 2020 (Poisson regression analysis; *p* = 0.043), indicating that the incidence of DRF reduced even after adjustment for the impact of population aging. In contrast, the age-adjusted incidence of DRF in males showed no significant trend (*p* = 0.90) (Fig. 2).

**Table 1:** Annual incidence of distal radial fracture per 100,000 population/year during 2011–2020

	**Population**			**Number of fractures**			**Annual incidence (95% CI)**		
**Years**	**Total**	**Male**	**Female**	**Total**	**Male**	**Female**	**Total**	**Male**	**Female**
2011	13,155	6242	6913	26	7	19	197.6 (134.9–289.6)	112.1 (54.3–231.5)	274.8 (176.0–429.3)
2012	12,855	6098	6757	35	6	29	272.6 (195.8–378.6)	98.3 (45.1–214.7)	429.2 (298.8–616.4)
2013	12,647	5994	6653	25	8	17	197.6 (133.9–291.8)	133.9 (67.6–263.4)	255.5 (159.5–409.2)
2014	12,553	5960	6593	32	11	21	254.9 (180.6–359.9)	184.6 (103.1–330.5)	318.5 (208.3–487.0)
2015	12,225	5806	6419	31	6	25	253.6 (178.6–359.9)	103.3 (47.4–225.5)	389.5 (263.8–575.0)
2016	12,029	5723	6306	19	5	14	158.0 (101.1–246.7)	87.4 (37.3–204.5)	222.0 (132.2–372.7)
2017	11,803	5653	6150	23	6	17	194.9 (129.9–292.4)	106.1 (48.6–231.6)	276.4 (172.6–442.7)
2018	11,547	5535	6012	24	6	18	207.8 (139.7–309.3)	108.4 (49.7–236.5)	299.4 (189.4–473.3)
2019	11,266	5409	5857	20	4	16	177.5 (114.9–274.2)	74.0 (28.8–190.2)	273.2 (168.2–443.8)
2020	10,982	5260	5722	23	9	14	209.4 (139.6–314.3)	171.1 (90.0–325.2)	244.7 (145.7–410.7)

^a^Population data were obtained from the government statistics in Japan. Populations for each year were evaluated on March 31 between 2011–2013 and on January 1 between 2014–2020

**Fig. 3:**
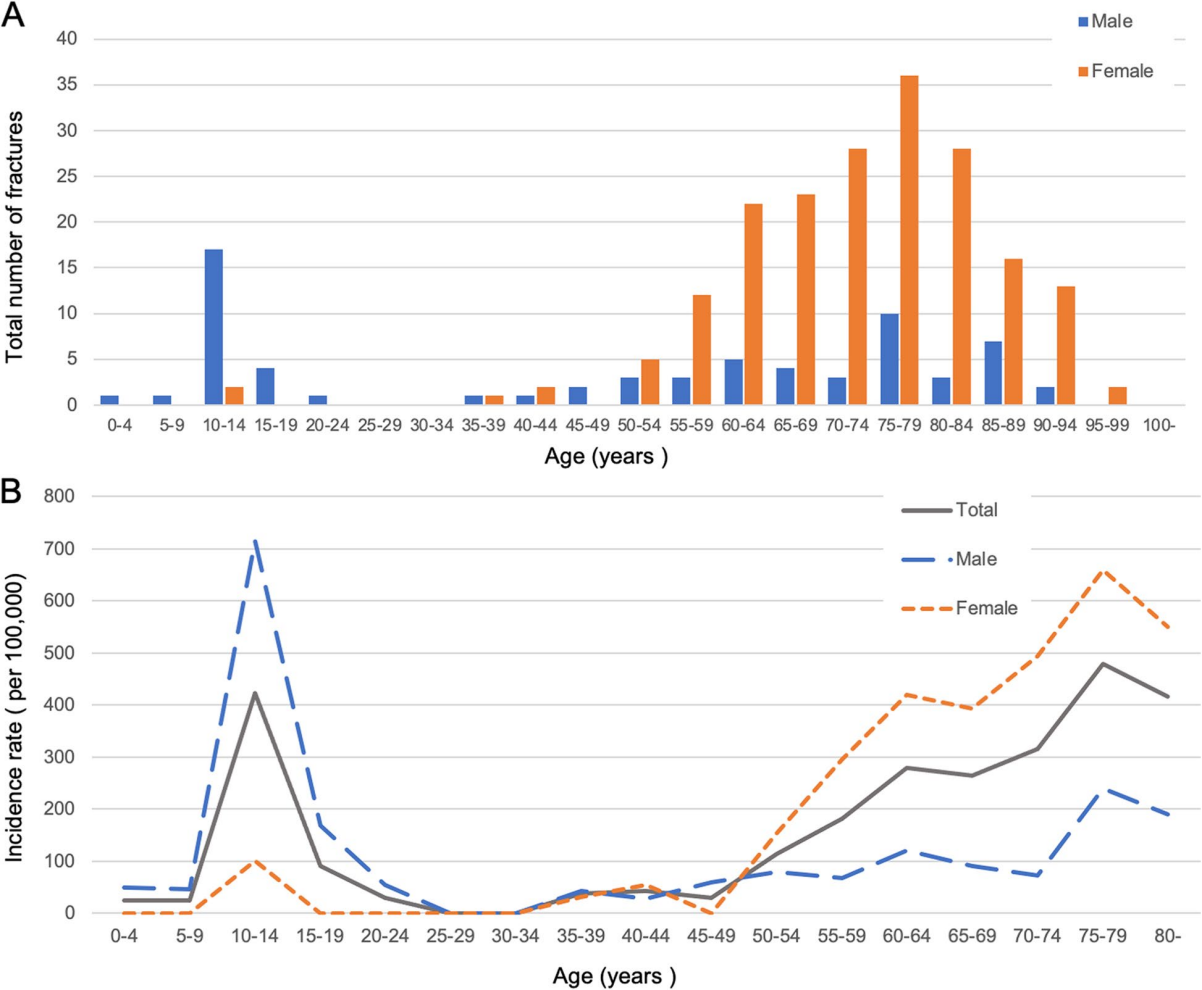
Age-specific incidence of distal radial fracture per 100,000 population/year. **A** Number of patients with distal radial fracture (DRF) in 5-year age groups; **B** Age-specific incidence of DRF in 5-year age groups

Figure 3 shows the distribution of the number of DRFs and age-specific incidence in 5-year age groups. There were sex differences in the distributions of both the number of DRFs and the incidence of DRF. The number of DRFs was bimodal, with peaks at 10–14 years of age for males and 75–79 years for females (Fig. 3A). The age-specific incidence in males was higher in younger patients (with the highest rate in teenagers), while the age-specific incidence in females was higher in older adults (with a marked increase in the incidence of DRF in women older than 50 years) (Fig. 3B).

### Injury characteristics

**Fig. 4:**
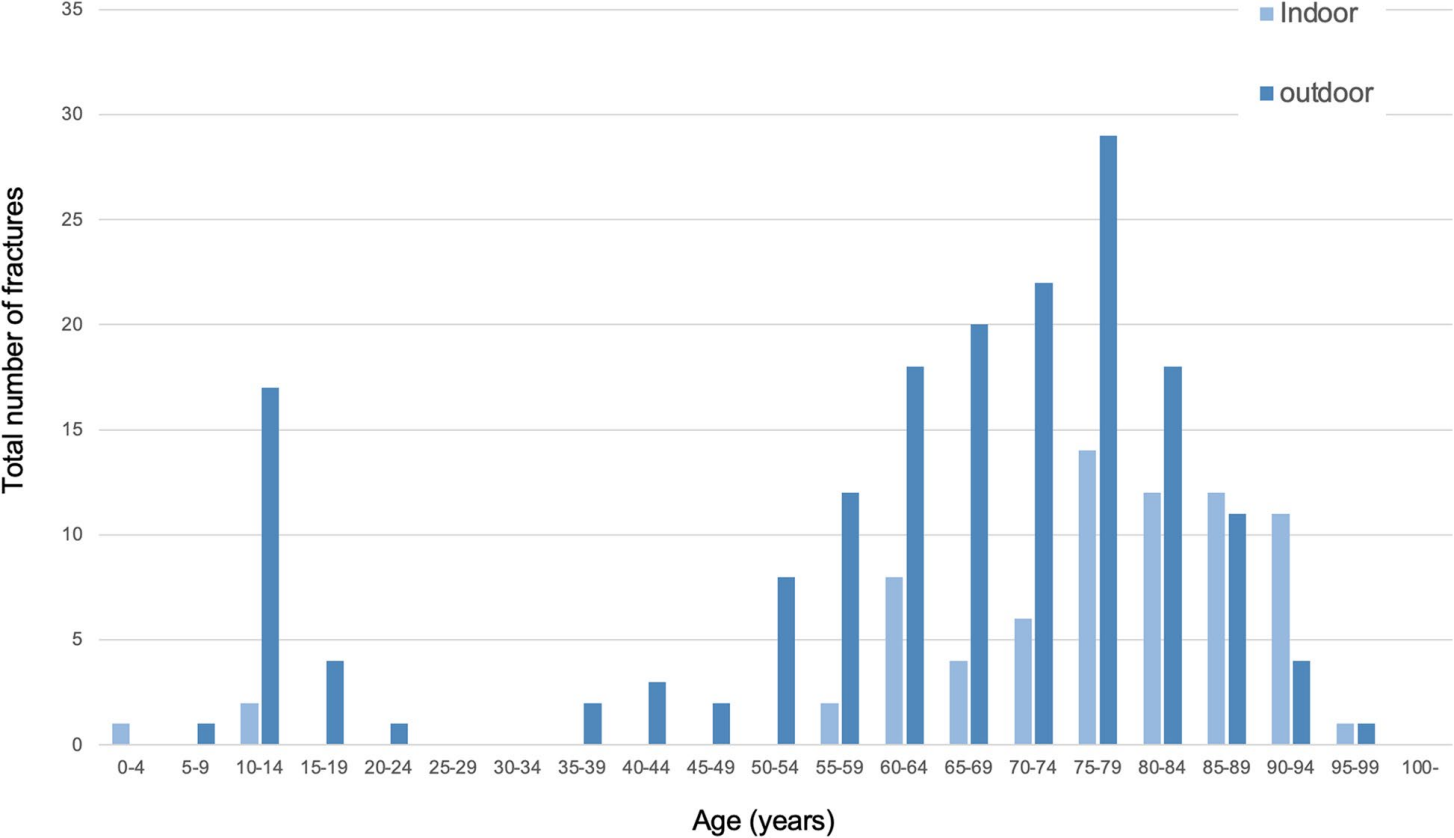
Injury locations of distal radius fracture in accordance with age

DRF was incurred outdoors in 173 of 258 patients (67.1%) (Fig. 4). Among the 85–89 year and older age groups, DRF tended to occur indoors rather than outdoors.

For the > 15 year group (Table 2), 85.3% of DRFs were caused by a simple fall (85.3%), followed by a fall from height (6.9%). However, for the ≤ 15 years group, the most common cause of DRF was sports injuries (50.0%), followed by traffic accidents (33.3%), showing that the injury cause greatly differed between younger and older age groups.

**Table 2:** Injury causes of distal radial fracture

	**Number (%)**		
**Injury causes**	**All age**	** ≤ 15 years of age**	** > 15 years of age**
Simple fall	199 (77.1)	1 (4.2)	198 (85.3)
Fall from height	19 (7.4)	3 (12.5)	16 (6.9)
Traffic accident	17 (6.6)	8 (33.3)	9 (3.8)
Sports injury	15 (5.8)	12 (50)	3 (1.3)
Crush injury	5 (1.9)	0 (0)	5 (2.1)
Other causes	2 (0.8)	0 (0)	2 (0.9)
No date	1 (0.4)	0 (0)	1 (0.4)
Total	258 (100)	24 (100)	234 (100)

**Fig. 5:**
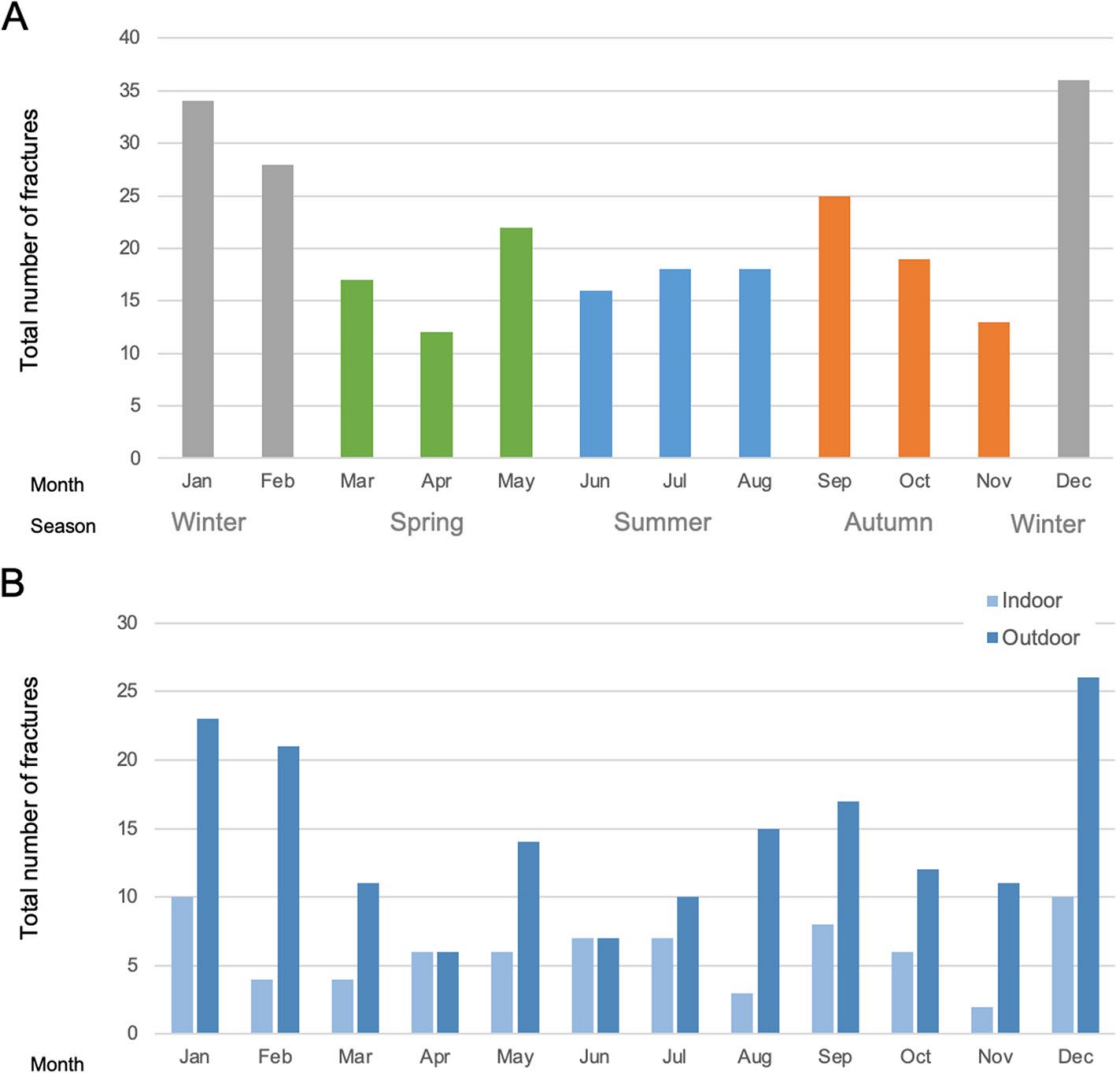
Seasonal differences in the incidence of distal radius fracture. **A** Total number of distal radius fractures (DRFs) by calendar month; **B** Total number of DRFs by calendar month in accordance with the injury location

There were seasonal differences in the incidence of DRF (Fig. 5). The number of patients with DRF was highest in the winter months, with an incidence of 14% (36/258) in December, followed by 13.2% (34/258) in January, and 10.9% (28/258) in February (Fig. 5A). DRF more commonly occurred outdoors than indoors throughout the year, but the proportion of outdoor injuries was particularly high in the winter season (Fig. 5B).

### Fracture classification and characteristics

The DRF was on the left side in 53% (137/258) of patients and the right in 47% (121/258). Table 3 shows the AO/OTA classification and characteristics of DRFs. In patients ≤ 15 years of age, the most common type of fracture was 23r-M/2.1 (41.7%; 10/24). In patients > 15 years of age, most DRFs were 2R3A2.1 (31.5%; 74/234) and 2R3A2.2 (31.9%; 75/234), and 78.7% (184/234) of patients were diagnosed with 2R3A fractures. All patients underwent radiographic examination at the initial fracture assessment. CT examination was additionally performed for 8.3% (2/24) and 65.4% (153/234) of patients aged ≤ 15 and > 15 years, respectively.

**Table 3:** Distal radius fracture classification and characteristics

AO/OTA classification	Number (%)	Mean age (SD)	Female (%)	CT scan use (%)
** ≤ 15 years of age**				
23r-M/2.1	10 (41.7)	12.0 (0.9)	2 (20)	2 (20)
23-M/2.1	4 (16.7)	8.5 (5.1)	0	0
23r-M/3.1	6 (25)	13.3 (1.9)	0	0
23-M/3.1	1 (4.2)	13.0	0	0
23r-E/2.1	3 (12.5)	13.0 (1.4)	0	0
Total	24 (100)	11.9 (2.9)	2 (8.3)	2 (8.3)
** > 15 years of age**				
A1	1 (0.4)	76.0	1 (100)	1 (100)
A2.1	74 (31.5)	71.1 (11.4)	57 (77)	56 (75.7)
A2.2	75 (31.9)	75.8 (11.8)	62 (82.7)	46 (61.3)
A2.3	3 (1.3)	81.3 (6.9)	3 (100)	0
A3.1	3 (1.3)	63.0 (5.1)	3 (100)	1 (33.3)
A3.2	20 (8.9)	76.2 (7.6)	18 (90)	10 (50)
A3.3	8 (3.4)	76.0 (8.2)	7 (87.5)	6 (75)
Total 2R3A	184 (78.7)	73.8 (11.3)	151 (82.1)	120 (65.2)
B1	2 (0.9)	47.0 (26.0)	1 (50)	1 (50)
B2	2 (0.9)	34.5 (18.5)	1 (50)	2 (100)
B3	0	—	—	—
Total 2R3B	4 (1.7)	40.75 (23.4)	2 (50)	3 (75)
C1	16 (6.8)	70.7 (13.7)	15 (93.8)	13 (81.3)
C2	0	—	—	—
C3	30 (12.8)	70.6 (13.1)	21 (70)	17 (56.7)
Total 2R3C	46 (19.6)	70.7 (13.3)	36 (78.3)	30 (65.2)
All	234 (100)	72.6 (12.8)	189 (80.8)	153 (65.4)

*Abbreviations*: *AO/OTA* Arbeitsgemeinschaft für Osteosynthesefragen Foundation/Orthopaedic Trauma Association, *CT* computed tomography

In patients ≤ 15 years of age, 29.2% (7/24) developed DRF with a concomitant ipsilateral distal ulnar fracture and 71.4% (5/7) had a distal diaphyseal fracture of the ulna. Among patients > 15 years of age with DRF, 36.8% (86/234) had a concomitant ipsilateral distal ulnar fracture, of which 80.2% (69/86) were ulnar styloid fractures. All patients ≤ 15 years of age with a DRF did not receive surgical treatment (i.e., received conservative treatment only). In contrast, 29.1% (68/234) of patients > 15 years of age received surgical treatment for DRF.

### Mortality

**Fig. 6:**
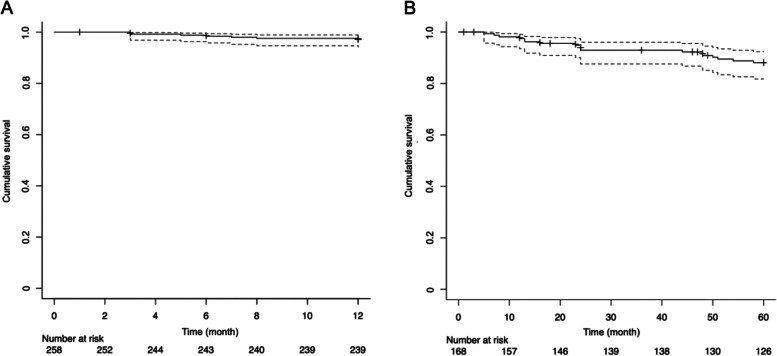
Kaplan–Meier survival curve with 95% confidence intervals. **A** Survival curves in one year for patients after DRF injuries during 2011–2020; **B** Survival curves in five years for patients after DRF injuries during 2011–2016. Upper and lower dotted line indicated the 95% confidence intervals

Of the total 258 patients, 92.6% (239/258) were followed up for 1 year after injury, while 2.3% (6/258) died during the 1-year period and 5.0% (13/258) could not be followed up for 1 year. The 1-year mortality rate after DRF injuries was 2.8% (Fig. 6A). Of the 168 patients who incurred a DRF between January 1, 2011, and December 31, 2016, 75.0% (126/168) were followed up for 5 years after injury, while 10.7% (18/168) died during the 5-year period and 14.2% (24/168) could not be followed up for 5 years. The 5-year mortality rate after DRF injuries was 11.9% (Fig. 6B).

## Discussion

Using regional population-based data, we identified the following epidemiological characteristics of patients with DRF in Japan. First, the incidence of DRF in our study was similar to that reported in other countries [7, 9, 11]. Second, the age-adjusted annual incidence of DRF among female patients showed a significant decreasing trend from 2011 to 2020. Third, the incidence of DRF was bimodal with a sex difference; among male patients the incidence was higher in younger patients (with a peak in teenagers), while among female patients the incidence was higher in older adults (and markedly increased after 50 years of age). These findings were also consistent with those reported in other countries [1, 3, 9, 17]. Fourth, DRF was more likely to occur during the snowfall season, which was consistent with the results of studies conducted in Northern Europe and Korea [4, 5, 8]. Fifth, nearly all patients with DRF were diagnosed with AO/OTA fracture types A and C, and 71% of those were treated conservatively, which may be affected by the large proportion of older patients in our study. Sixth, the 1- and 5-year mortality rates among patients with DRF were similar to those reported in other countries [4, 21, 22], and were lower than the death rates among patients with other common osteoporotic fractures such as hip and spine fractures [23].

In our study, the age-adjusted incidence rates of DRF showed a significant decreasing trend over a 10-year period among female patients. The findings of previous studies suggest that this decrease may be due to the establishment of standard osteoporosis treatment for older women, which reduces the incidence of osteoporotic fractures [3, 9, 24]. Another potential reason for the decrease is the effective implementation of public health measures for fall prevention among older people. For example, previous studies have reported that fall prevention measures in the winter season with snow and ice conditions prevent the occurrence of DRF and other osteoporotic fractures among older adults [9, 24–27]. In Hokkaido, public health measures have been implemented to prevent falls in snow and ice conditions in the winter season, which may have contributed to the declining incidence of DRF in our study [28].

Our results indicated that the age-specific incidence of DRF was bimodal with peaks in 10–15-year-old males and 70–79-year-old females. These bimodal age patterns in DRF incidence are consistent with the findings of studies conducted in other countries and with a study conducted in Sado City in Japan [1, 3, 9, 17]. Our results showed that the incidence of DRF increased with age in women older than 50 years, but decreased after 80 years of age. This may be because patients older than 80 years may find it more difficult to protect themselves with their hands in the event of a fall, resulting in hip fracture or proximal humerus fracture rather than DRF [17, 29]. In contrast, the DRF incidence tended to peak in males aged 10–14 years. This is consistent with previous findings and suggests that physical activity such as sports may be associated with DRF in younger patients [3, 30].

Previous studies have reported that the incidences of AO/OTA fracture types A, B, and C range from 54 to 67%, 9% to 14%, and 23% to 32%, respectively [4, 11, 31]. In our study, the proportions of AO/OTA fracture types A, B, and C were 78.7%, 1.7%, and 19.6%, respectively, indicating that a larger proportion of patients had type A fracture while a smaller proportion of patients had fracture types B and C compared with the proportions reported in other studies [4, 11, 31]. Previous studies have demonstrated that type B and C fractures are more common in younger patients than type A fractures [4, 11, 31]. Thus, our results were reasonable because our cohort included a small proportion of younger patients. The higher proportion of type A fractures may be affected by the large proportion of older adults who developed DRF by a simple fall.

The present study has some limitations. First, our study had a small sample size compared with previous studies conducted in Japan [12, 15–17]. However, our findings were largely consistent with those of previous studies conducted within and outside of Japan [1, 4, 5, 7–9, 11, 17, 21, 22]. Furthermore, the 10-year study period in our study was longer than the study period of previous studies conducted in Japan [12, 15–17]. Second, some patients with DRF might have been treated conservatively by non-orthopedic surgeons and judo therapists, or might have been diagnosed and treated in medical facilities located outside of our study setting. Third, our study was a single-center study, and the diagnosis and treatment might differ from other medical facilities. Fourth, this study was conducted in a cold and snowy region of Japan, which may have a higher incidence of DRF in winter than other warmer regions. Furthermore, population aging may also affect the incidence of DRF and the death rates. For these reasons, our results might not represent the general population of Japan. Fifth, we did not have access to information on osteoporosis treatments. Sixth, 5% and 14% of patients were not followed up for 1 and 5 years, respectively (missing cases), which may underestimate or overestimate the mortality rates. Finally, the coronavirus disease 2019 pandemic affected the behavior of people in our study setting by reducing the frequency of outings during 2020, which may have affected our results.

In conclusion, using regional population-based data, we identified the epidemiological characteristics of patients with DRF. The annual incidence of DRF ranged from 158.0 to 272.6 per 100,000 population/year during 2011–2020. The age-adjusted annual incidence of DRF among female patients showed a significant decreasing trend from 2011 through 2020. Osteoporotic management might have contributed to the declining incidence of DRFs in females. Further research is warranted to investigate the effect of osteoporotic treatment on the incidence of DRFs. The incidence of DRF was bimodal and was highest in teenagers in the male population and in older adults in the female population. This suggests the need for public health measures to prevent sports injuries in young males and to prevent falls in older females.

## Data Availability

The datasets generated and/or analyzed during the current study are not publicly available because of the risk of identifying patients using information such as age, sex, date of injury, and residence of the patients, but are available from the corresponding author on reasonable request.

## Abbreviations

DRF: Distal radius fracture
CT: Computed tomography
AO/OTA: Arbeitsgemeinschaft für osteosynthesefragen foundation/orthopaedic trauma association
SD: Standard deviation
CI: Confidence interval
